# Efficacy and safety of doxercalciferol (1α hydroxyvitamin D_2_) on spine and femur bone mineral density in postmenopausal osteopenic women

**DOI:** 10.3389/fendo.2026.1640045

**Published:** 2026-07-23

**Authors:** J. C. Gallagher, Richard Mazess

**Affiliations:** 1 Bone Metabolism Unit, Creighton University School Medicine, Omaha, NE, United States; 2 Department Medical Physics, University of Wisconsin, Madison, WI, United States

**Keywords:** bone mineral density, doxercalciferol, osteoporosis, randomized clinical trial, Vitamin D2 analog 1α hydroxyvitamin D2

## Abstract

**Background:**

The use of small doses of vitamin D_3_ analogs or metabolites is common in the prevention of renal osteodystrophy. Their use in the treatment of osteoporosis has been limited by their potent adverse effects, causing hypercalcemia and hypercalciuria. We evaluated a vitamin D_2_ analog, i.e., 1α-hydroxyvitamin D_2_ (doxercalciferol), that has a lower incidence of adverse effects.

**Methods:**

In a 1-year pilot randomized double-blind, placebo-controlled study, 55 postmenopausal women (aged 60–71 years) with osteopenia were treated for a year with either doxercalciferol or placebo, and 41 women completed an optional second year. The dose of 1 µg was increased by 1 µg at weekly intervals to a maximum of 5 µg. The daily dose of doxercalciferol averaged 4.1 µg after 12 months and 3.6 µg after 24 months. Bone mineral density (BMD) measurements by dual-energy X-ray absorptiometry (DEXA) of the spine and femoral neck were obtained at 6-month intervals for 24 months. The serum and urine calcium levels were measured frequently for safety.

**Results:**

In the primary intention-to-treat (ITT) analysis at 12 months, there was no significant difference between the two groups for femoral neck and spine BMD. At 24 months, there was a significant difference in favor of doxercalciferol for femoral neck BMD, with the treatment/placebo difference being 1.70% (*p* < 0.047); for spine BMD, the treatment/placebo difference was 1.1% (*p* = 0.36). In the per-protocol analysis at 12 months, the doxercalciferol/placebo difference for femoral neck was 0.5% (*p* = 0.497) and for spine BMD was 1.6% (*p* = 0.071). At 24 months, the doxercalciferol/placebo difference for femoral neck BMD was 2.2% (*p* < 0.017) and for spine BMD was 2.4% (*p* < 0.041). Over 24 months, there were five mild hypercalcemia events, 2.54 mmol/L (>10.2 mg/dl), in five patients. The mean 24-h urine calcium increased by 3.75 mmol (150 mg), with 16 episodes of hypercalciuria, 10 mmol (>400 mg), in 12 patients.

**Conclusions:**

The results show that doxercalciferol treatment showed no difference from the placebo group on bone loss at 12 months; however, at 24 months, doxercalciferol showed significant prevention of bone loss from the femoral neck and spine.

**Clinical Trial Registration:**

https://clinicaltrials.gov/, identifier NCT07660536.

## 1 Introduction

Vitamin D in the diet or from the sun is transported to the liver, where it undergoes 25 hydroxylation [25-hydroxyvitamin D, 25(OH)D]. This metabolite, which is not biologically active, is transported to the kidney for further hydroxylation at the 1-position to form 1,25 dihydroxyvitamin D_3_ [1,25(OH)_2_D_3_] (calcitriol), the biologically active hormone. Neither vitamin D3 nor 25-hydroxyvitamin D_3_ [25(OH)D_3_] acts on intestinal calcium absorption; however, it is calcitriol that is the main stimulus to calcium absorption (1). Calcitriol metabolism is regulated by the parathyroid hormone (PTH) in a negative and positive feedback loop (2) and by FGF23 in a positive–negative feedback loop (3). It is well known that age is associated with reduced calcium absorption (4) and lower levels of serum 1,25(OH)_2_D3 (5). Despite the secondary hyperparathyroidism associated with aging (6, 7) or the direct stimulation of the kidney by intravenous PTH (8), serum 1,25(OH)_2_D_3_ does not increase, but continues to decline with age probably due to the aging kidney. Patients with osteoporosis also have malabsorption of calcium (9, 10) and reduced levels of 1,25(OH)_2_D_3_ (10). Surprisingly, earlier studies showed that plain vitamin D_3_ or D_2_ did not increase calcium absorption until the daily dose reached 10,000 IU (9). Thus, there is a rationale for the treatment of elderly people and patients with osteoporosis with physiologic doses of calcitriol to improve calcium absorption and reduce secondary hyperparathyroidism. However, when given orally, calcitriol has a potent effect on calcium absorption, and to avoid hypercalcemia, the calcium intake should be restricted to 600 mg daily (11, 12).

To avoid direct stimulation of the intestine, vitamin D receptor (VDR) led to the development of prodrugs. Prodrugs such as alfacalcidol (1α-hydroxyvitamin D_3_) and doxercalciferol (1α-hydroxyvitamin D_2_) already have the hydroxyl group in the 1-position and need only hydroxylation in the 25-position by the liver to produce their respective metabolites, i.e., 1α,25(OH)_2_D_3_ and 1α,25(OH)_2_D_2_. Thus, after oral ingestion, prodrugs will not become active until they pass through the liver. Earlier studies using alfacalcidol in daily doses of 0.5–2.0 µg showed increased calcium absorption and improved calcium balance (13–15). In a comparative study, 1 µg alfacalcidol increased calcium absorption, whereas daily vitamin D at 1,000 IU had no effect (16). Another analog used in Asia is eldecalcitol (1,19-hydroxyvitamin D_3_) at a daily dose of 0.75 µg, which has a similar effect on calcium absorption to that of 1 µg alfacalcidol (17).

Earlier placebo-controlled studies of synthetic calcitriol (Rocaltrol) did not consistently show a reduction in fractures (18–20); however, the total number of patients in these studies was very small, 83 on placebo and 80 on calcitriol, and underpowered for fracture analysis. In a 3-year phase 4 study of 622 women in a primary care setting, a calcitriol dose of 0.25 µg twice daily was compared with a control group given daily calcium supplements of 1,000 mg. A significant reduction in fractures was observed in both the spine (64%) and peripheral bones (50%) over 3 years (21).

Both alfacalcidol and calcitriol prevent bone loss and reduce fracture rates (21–23). When the two analogs were analyzed separately, alfacalcidol was found to be superior to calcitriol (24). The daily therapeutic dose window of calcitriol is narrower (0.5–0.75 µg) on a normal calcium intake of 600–700 mg daily (11, 12) than that of alfacalcidol (0.5–2.0 µg) (13–15). Eldecalcitol at 0.75 µg was more effective in reducing fractures than the 1-μg dose of alfacalcidol (25); however, an equivalent dose for alfacalcidol may be 1.5 μg. Similar to alfacalcidol and calcitriol, the window of efficacy *versus* toxicity for eldecalcitol is also limited. During the early dose–response studies, the incidence of hypercalcemia was 6% for the 0.75-µg/day dose and was 23% for 1 µg/day (26–28).

Plain vitamin D_3_ in doses ranging from 400 to 4,000 IU did not increase the bone mineral density (BMD) or reduce the bone resorption markers (29). In a 5-year placebo-controlled trial of vitamin D_3_ at 2,000 IU daily in 25,000 men and women, there was no reduction in fractures (30). However, the majority of patients (65%) also had normal serum 25(OH)D and therefore may not respond to vitamin D. However, in a subgroup analysis of 400 subjects with serum 25(OH)D levels <12 ng/ml, there were no differences in the fracture rates. This may be due to the aging kidney not being able to convert 25(OH)D to 1,25-dihydroxyvitamin D [1,25(OH)_2_D].

Doxercalciferol, the corresponding D_2_ prodrug, has similar biological effectiveness as alfacalcidol (31), but has lower toxicity in animals (32), which may be due to the suppression of endogenous 1,25(OH)_2_D (33). The vitamin D_3_ analogs all easily develop hypercalcemia with doses up to 1 µg daily if the calcium intake is greater than 800 mg daily. Due to a previous dose-ranging study of doxercalciferol (1α-OH-D_2_) showing minimal effects on the serum and urine calcium up to a dose of 5 µg (34), there has been renewed interest in examining the efficacy and safety of 1α-OH-D_2_ in preventing age-related bone loss. In this study, BMD of the femoral neck and spine was the primary outcome, while safety measurements of serum and urine calcium were the secondary outcomes.

## 2 Methods

### 2.1 Subject selection

Postmenopausal women, between ages 60 and 78 years, were recruited from clinics and through advertisements. Participants were screened to confirm normal baseline serum and urinary biochemical parameters. Bone mineral density of the proximal femur and L2-3 vertebrae was measured by dual-energy x-ray absorptiometry (DEXA;LUNAR DPX, GE Healthcare, Madison, WI, USA). The inclusion criterion was spine BMD between 0.70-1.05g/cm2 corresponding to a T score between -2.5 and -1.0. Lateral radiographs of the lumbar and thoracic spine, kidney, ureter, and bladder were performed, as well as determinations of serum PTH and 25(OH)D. The exclusion criteria encompassed significant medical disorders, recent use of medications known to affect bone or calcium metabolism, history of renal calculi, previous prolonged hospitalization or other extended immobilization, radiographic evidence of more than three non-traumatic vertebral fractures, previous non-traumatic hip fracture, and recent changes in exercise habits. Radiographs of the kidney, ureter, and bladder were performed at baseline and at 12 and 25 months for the presence of renal calculi.

The protocol and informed consent form were reviewed and approved by the Institutional Review Board at Creighton University and by the Research Review Board of the Food and Drug Administration (FDA) (IND 31,423). Informed consent was obtained from all subjects. This study started in November 1989 and was completed in April 1992, before the Clinical Trials registry (clin.gov) went public in February 2000.

Qualifying volunteers underwent a full physical examination. Based on the screening examinations, 56 subjects who met the inclusion criteria were selected.

### 2.2 Study design

The study was conducted according to a double-blind, placebo-controlled design. In the first phase, each subject was assigned at random to one of two treatment groups for 52 weeks. In the second phase, the blinded study was continued as a participant option for another 52 weeks. One treatment group received up to a 104-week course of oral therapy with doxercalciferol; the other group received a matching placebo therapy. On enrollment, subjects were interviewed by a registered dietician to obtain 7-day diet histories. The subjects were then instructed to self-select diets containing 700–900 mg calcium (without using calcium supplements) and to adhere to such diets for the duration of the study. All subjects provided blood and urine samples for routine tests and underwent a measurement of fractional calcium absorption from the small intestine prior to beginning treatment. During the treatment period, subjects in the groups ingested doxercalciferol or placebo at an initial daily dosage of 1.0 µg and increased the dosage to 2.0, 3.0, and 4.0 µg in each of the following weeks, to a maximum dosage of 5.0 µg. The dosage for any given subject was increased in this manner until the daily urinary calcium excretion increased to more than 7.5 mmol/L (300 mg), at which point the dosage was held at that level long term. Drug dosing was suspended during episodes of hypercalcemia, i.e., 2.54 mmol/L (>10.2 mg/dl), or hypercalciuria, i.e., 10 mmol/L (>400 mg), and restarted at a dosage that was 0.5 μg lower than the previous dose. Dosing was reduced in increments of 0.5 μg in response to persistent hypercalcemia or mild hypercalciuria. Compliance with the prescribed dosing was monitored by weekly counts of capsules returned and by subject interviews. General compliance to the calcium-regulated diets was monitored at regular intervals using daily food records and by interviews with each subject by the dietician. Blood and urine chemistries were monitored in all subjects at weekly intervals during the initial 6 weeks of the treatment period and at quarterly intervals thereafter. Fasting blood and urine samples were obtained 24 h post-dosing, just before the next scheduled dose, while daily urine samples were collected between consecutive dosings together with daily diet diaries. BMD measurements of the L2–L3 vertebrae and femoral neck were obtained at 6-month intervals. Lateral X-rays of the lumbar and thoracic spine; X-rays of the kidney, liver, ureter, and bladder; and measurements of the intestinal calcium absorption were obtained at annual intervals. All medical events noted by the staff or complaints expressed by the subjects were recorded, stating the type, onset, duration, severity, relationship to the test drugs, required treatment, and outcomes.

### 2.3 Procedures

Routine blood and urine assays [e.g., complete blood count (CBC) with differential, serum chemistries, urinalysis with microscopic analysis, and urine chemistries] were completed according to laboratory standard operating procedures. More specialized blood and urine assays were completed as indicated: serum and urine calcium with the Abbott VP Bichromatic Analyzer (Abbott Laboratories, Abbott Park, IL, USA); serum ionized calcium with Radiometer (Copenhagen, Denmark); serum calcifediol [25(OH)D] using a competitive binding assay (35); serum doxercalciferol and lα,25-(OH)D_2_ or 1,25-dihydroxyvitamin D_2_ using a radioreceptor assay after high-performance liquid chromatography (36, 37); serum intact PTH with a radioimmunoassay (38) using a mid-molecule antiserum (a gift from R.D. Hesch, Hanover, Germany); serum osteocalcin by adapting previously published immunoassay procedures (39); urine phosphorus and creatinine using colorimetric assays; and urine hydroxyproline with the Kivirikko–Prockop method. Calcium absorption was measured with a single isotope technique using radiocalcium Ca^45^ in a 100-mg calcium carrier, while the fractional calcium absorption was calculated as described previously on the 3- and 4-h samples (10). The DEXA densitometer for BMD was inspected by the manufacturer at quarterly intervals to verify proper functioning and was calibrated daily using an internal standard and weekly using external spine and femur phantoms certified by the manufacturer. Vertebral fractures were diagnosed from the lumbar spine, as previously described (40).

### 2.4 Test drugs

Bulk doxercalciferol was synthesized according to previously described methods (41, 42), formulated in fractionated coconut oil containing 0.03% butylated hydroxyanisole and encapsulated in soft gelatin capsules with strengths of 0.5 and 1.0 µg. Matching placebo capsules for doxercalciferol contained an equivalent weight of fractionated coconut oil. Active and placebo capsules were identically packaged in opaque plastic bottles to prevent possible photolysis. For the statistical plan, randomization of subjects to the treatment groups according to a randomization code was prepared by an independent statistician using the Statistical Analysis System (SAS). No staff involved in the study had access to the randomization code, except for the principal investigator who could determine at his discretion a subject’s treatment assignment by opening a sealed opaque envelope for the subject; otherwise, the principal investigator did not break the randomization code until all analyses of the DEXA scans had been completed and analyzed by the statistician. As an additional precaution, staff members who performed analyses of the DEXA scans and lateral radiographs did not have access to other data obtained from the subjects.

### 2.5 Statistical analysis

All subjects who commenced test drug were evaluable for the intention-to-treat (ITT) analysis. Subjects were considered evaluable for the per-protocol (PP) analysis if they met the selection criteria at enrollment, commenced test drug dosing, and consumed at least 85% of the prescribed dosage. The variables analyzed for primary evidence of the efficacy of the test drug were BMD of the L2–L3 vertebrae and femoral neck, with the baseline values for BMD defined as the mean of all measurements obtained within 120 days prior to starting the test drug and the intestinal calcium absorption. Safety measures included serum and urine calcium, blood urea nitrogen (BUN), creatinine clearance, and fasting urinary hydroxyproline. Baseline values for all other parameters were defined as the last observations made prior to administering the test drug. Treatment groups were compared with respect to the baseline values using a *t*-test or a Wilcoxon two-sample test, while post-baseline values used a paired *t*-test or a Wilcoxon one-sample test. In addition, the ITT analysis included an endpoint analysis using the data collected at the last visit for each subject. The BMD results were analyzed over the course of test drug therapy using Fisher’s exact test and two-way analysis of variance. Increases in the frequency of adverse experiences were similarly examined. Any adverse experiences found to occur with frequency greater than 5% were examined using the Kaplan–Meier method to estimate the survival curves associated with the time to the occurrence of the adverse effect. Inter-group differences were assessed using the log-rank procedure and the generalized Wilcoxon procedure. All computations were performed using SAS, with statistical significance declared for the two-sided *p*-value 0.05 (43). In a *post-hoc* calculation, with 56 subjects (group sizes of 28 and 27), there is 80% power at 52 weeks to detect a percent change difference of 2.3% between the randomized groups with a standard deviation for both groups of 2.7% (alpha = 0.025 using a two-sided equal variance *t*-test). In addition, with 41 subjects (group sizes of 21 and 20), there is 80% power at 104 weeks to detect a percent change difference of 2.6% between the randomized groups with a standard deviation for both groups of 2.7% (alpha = 0.025 using a two-sided equal variance *t*-test). The type I error rate was adjusted in these calculations for comparisons at 52 and 104 weeks.

## 3 Results

### 3.1 Participant enrollment

There were 60 subjects initially enrolled, with four withdrawn before randomization due to a previous history of non-insulin-dependent diabetes, leaving 56 randomized study subjects, 29 of whom on doxercalciferol and 27 on placebo. One subject on doxercalciferol dropped out during the third treatment week, complaining of transient blurred vision, a symptom that she had reported prior to enrollment into the study. At termination, she had been on a dose of 3.0 µg/day of doxercalciferol. Thus, 55 subjects (doxercalciferol = 28, placebo = 27) completed the initial 52-week treatment period. **A total of** 42 subjects (doxercalciferol = 21, placebo = 21) re-enrolled in an optional 1-year extension of the study, during which time they continued test drug dosing as before without interruption. One patient on doxercalciferol dropped from the study in year 2 for personal reasons. Thus, 41 subjects (doxercalciferol = 20, placebo = 21) completed the second year (**Table 1**).

**Table 1:** Participant enrollment, randomization, and follow-up.

Subject randomization	1α-OHD_2_	Dropouts	Placebo	Dropouts
Baseline	29		27	
End year 1	28	1	27	0
Start year 1	21	7^a^	21	6^a^
End year 2	20	1	20	1

^a^ Did not re-enroll in year 2.

### 3.2 Participant characteristics

The participant characteristics at baseline and at the end of years 1 and 2 on doxercalciferol and placebo are shown in **Table 2**. There were no statistical differences between the groups at baseline.

**Table 2:** Baseline characteristics and changes at the end of years 1 and 2 on doxercalciferol/placebo mean *T* scores at baseline.

	Placebo baseline	Placebo end of year 1	Placebo end of year 2	1α-OHD_2_ baseline	1α-OHD_2_ end of year 1	1α-OHD_2_ end of year 2
	Mean (SD)	Mean (SD)	Mean (SD)	Mean (SD)	Mean (SD)	Mean (SD)
Age (years)	65.5 (3.1)			65.6 (3.6)		
Years since menopause	17.6 (6.2)			18.8 (7.3)		
Height (in.)	63.3 (2.0)	63.0 (2.0)	63.0 (1.7)	62.9 (2.0)	62.9 (1.7)	63.2 (1.8)
Weight (lbs)	147.(27)	148(21)	148 (21)	143(17)	144 (18)	152 (20)
Vertebral fractures (*n*)	0.2 (0.5)	0.3 (0.6)	0.3 (0.5)	0.2 (0.6)	0.3 (0.6)	0.3 (0.7)
Dietary calcium intake (mg)	699 (247)		742 (86)	848 (381)		772 (89)
Dietary calcium compliance (%)		90 (19)	98 (4.8)		86 (18)	95.60 (23.57)
Mean daily dose 1-alphaD_2_ (µg)		4.81 (0.86)	4.76 (0.98)		4.16 (1.22)	3.61 (1.52)
Mean compliance 1-alphaD_2_ (%)		97 (5)	97 (3)		94 (7)	95 (6)
Spine BMD (g/cm^2^)^a^	0.929 (0.095)			0.937 (0.078)		
Spine BMD (% change ITT)		99.5 (2.6)	99.1 (3.1)		100.6 (3.7)	100.2 (4.6)
Spine BMD (% change PP)		99.5 (2.6)	98.7 (2.6)		100.1 (3.6)	101.1 (4.2)*,^b^
Femur neck BMD (g/cm^2^)	0.728 (0.074)			0.776 (0.092)		
Femur neck BMD (% change ITT)		100.3 (3.12)	98.3 (2.70)		100.9 (2.64)	100.0 (2.62)^b^
Femur neck BMD (% change PP)		100.2 (3.13)	98.3 (2.76)		100.7 (2.57)	100.5 (2.41)**
Serum calcium (mmol/L)	2.24 (0.05)	2.35 (0.06)	2.39 (0.06)	2.27 (0.07)	2.40 (0.1)	2.35 (0.08)
Serum ionized calcium (mmo/L)	1.22 (0.03)	1.12 (0.03)	1.24 (0.03)	1.22 (0.03)	1.25(0.06)	1.26 (0.03)
Serum phosphorus (mmol/L)	1.12 (0.24)	1.16 (0.14)	3.709 (0.42)	1.2 (0.16)	1.15 (0.1)	1.16 (0.16)
Serum creatinine (μmol/)	66.3 (11.5)	66.3 (7.09)	0.80 (0.12)	70.7 (16.80	74.3 (16.8)	74.3 (15)
Serum osteocalcin (ng/ml)	3.88 (1.22)	3.99 (1.03)	4.40 (1.32)	3.909 (1.36)	4.07 (1.22)	4.34 (1.31)
Serum PTH (pg/ml)	117 (58)	128 (69)	176 (83)	138 (60)	110 (54)	145 (73)
24-h urine calcium (mmol/L)	4.15 (1.82)	3.62 (1.6)	3.27 (1.35)	3.7 (2.17)	6.6 (1.77)	7.67 (2.7)
24-h urine creatinine (μmol/L)	86,209 (16,044)	79,401 (15,166)	78,428 (21,746)	87535 (23,784)	86,366 (21,481)	87,624 (19,717)
Creatinine clearance (ml/min)	93 (24)	85 (19)	78 (22)	85 (26)	85 (25)	85 (24)
24-h urine OHPR/Cr	0.024 (0.007)	0.026 (0.007)	0.024 (0.007)	0.024 (0.006)	0.023 (0.008)	0.023 (0.006)
Serum 1,25 (OH)_2_D_3_ (pmol/L)	100.7 (28.1)	100.6 (25)	90.865 (21.7)	89.4 (26.5)	44(27.5)	46.6 (32.9)
Serum 25(OH)D (nmol/L)	73.63 (26.7)	73.88 (31)	67.14 (24.2)	75.13 (27.5)	69.24 (21.22)	63.4 (18.0)
Serum total 1,25(OH)_2_D (pmol/L)	114 (26.7)	113,6 (24.5)	99.4 (21.2)	100.8 (29.4)	148.6 (44.9)	148.1 (577.1)
Serum 1,25(OH)_2_D_2_ (pmol/L)	13.45 (15.3)	8.8 (5.7)	8.6 (10.5)	11.4 (10.8)	105.6 (47.4)	101.1 (6.7)
Calcium absorption (% AD/L)	2.89 (1.0)	2.96 (0.8)	2.82 (0.68)	2.92 (1.14)	3.97 (1.12)+	3.5 (0.95)+
Intention to treat (*n*)	27	27	21	29	28	20
Per protocol (n)	26	26	20^c^	25	25	17^c^,^d^

Spine BMD, −2.1 (1.1); femoral neck BMD, −1.9 (0.8).

Per protocol (PP): **p = 0.017 (femoral neck); *p = 0.041 (PP spine).

^a^ Statistical tests: 1α-OHD_2_ group compared with placebo.

^b^ Intention-to-treat (ITT) analysis: *p* = 0.047 (femoral neck); *p* = 0.360 (spine).

^c^ Three patients with hyperparathyroidism were excluded from the PP analysis (two doxercalciferol and one placebo).

^d^ One patient on doxercalciferol in whom no drug was detected in multiple blood samples. T scores, Spine BMD,-2.1 (1.1);femoral neck BMD -1.9(0.8). +, p=0.01.

### 3.3 Compliance for doxercalciferol and dietary calcium

Dose compliance for doxercalciferol averaged 94% at the end of year 1 and 93% at the end of year 2. Compliance in the placebo group averaged 97% at the end of year 1 and 98% at the end of year 2. At the end of the dose escalation phase, the daily doxercalciferol dose averaged 4.52 μg in week 5, 4.16 μg at 12 months, 3.63 μg at 18 months, and 3.61 μg at 24 months. In the placebo group, the equivalent doses were 4.84 μg at week 5, 4.81 μg at 12 months, 4.76 μg at 18 months, and 4.76 μg at 24 months. Dietary calcium compliance averaged 86% at 12 months and 99% at 24 months (mean calcium intake, 772 mg/day) in the doxercalciferol group. Compliance in the placebo group averaged 90% at 12 months and 98% at 24 months (mean calcium intake, 740 mg/day).

### 3.4 Biochemical results

#### 3.4.1 Serum calcium

**Figure 1:**
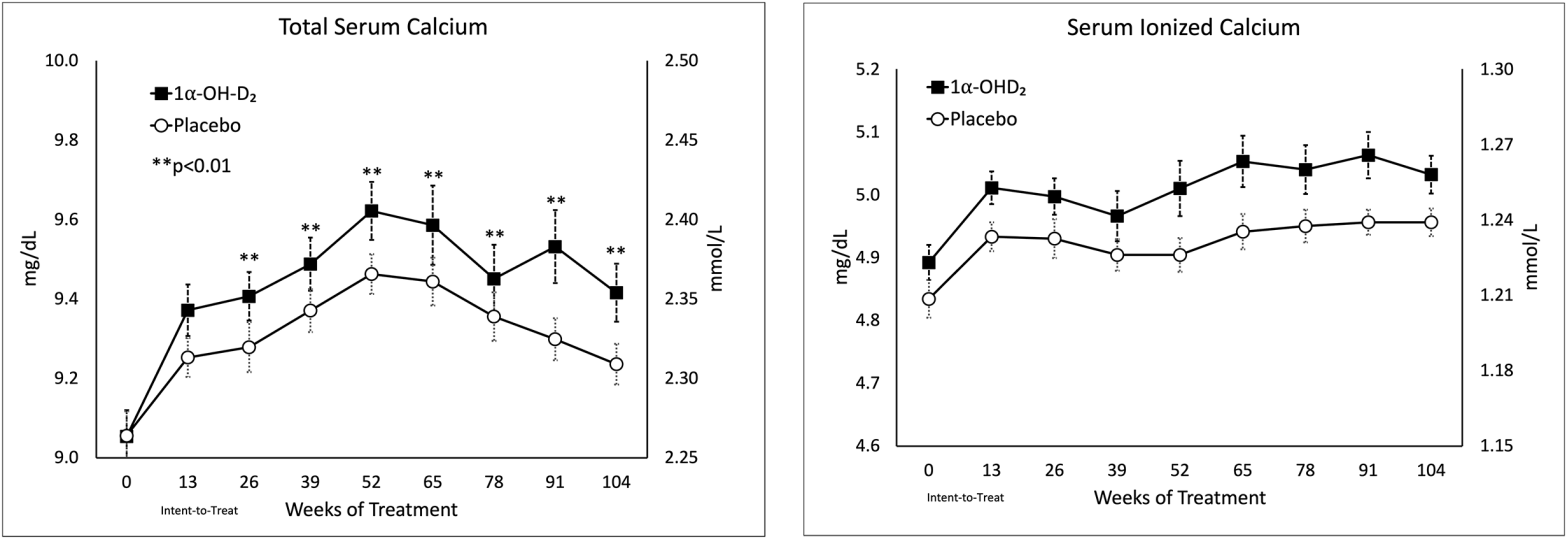
Total and ionized serum calcium on doxercalciferol or placebo. **p<0.01.

The mean serum calcium (in millimoles per liter) increased from a baseline of 2.27 to 2.40 at 12 months and to 2.35 by 24 months in the doxercalciferol group (**Table 2**; **Figure 1**). In the placebo group, the baseline serum calcium averaged 2.24 and increased to 2.36 by 12 months and 2.33 by 24 months. The weekly and monthly values are shown in **Figure 1**. There were two single episodes of mild hypercalcemia in doxercalciferol patients during the first year. In the second year, three other subjects had single episodes of mild hypercalcemia, with serum calcium levels of 2.68, 2.57, and 2.62. An episode of clinically significant hypercalcemia (2.99 mmol/L) occurred during an intercurrent illness in one subject, but which resolved quickly. In year 1, there were 2 out of 311 (0.64%) measurements of serum calcium that increased above the normal range. In year 2, there were 3 out of 84 (3.6%) measurements that increased above the normal range. In the placebo group, one patient had an increased value above normal (1 of 297 tests) in year 1, but none in year 2 (0 of 84 tests). Subjects on doxercalciferol had average serum calcium values approximately 0.04 mmol/L higher than those on placebo.

#### 3.4.2 Serum ionized calcium

The weekly changes in the values for serum ionized calcium (in millimoles per liter) are shown in **Figure 1**. One patient had one elevated total calcium value of 2.55 out of 11 total samples, but had five slightly high ionized calcium values ranging from 1.31 to 1.42. The serum PTH was elevated: she probably had mild hyperparathyroidism. She discontinued the study after 1 year. Another patient had 15 values of normal serum calcium over 2 years, but an elevated ionized calcium in all 15 samples (1.31–1.36). She had an elevated PTH value consistent with primary hyperparathyroidism. In the placebo group, there were no increased ionized calcium values in year 1, while one increased in year 2 (1 of 84 tests).

**Figure 2:**
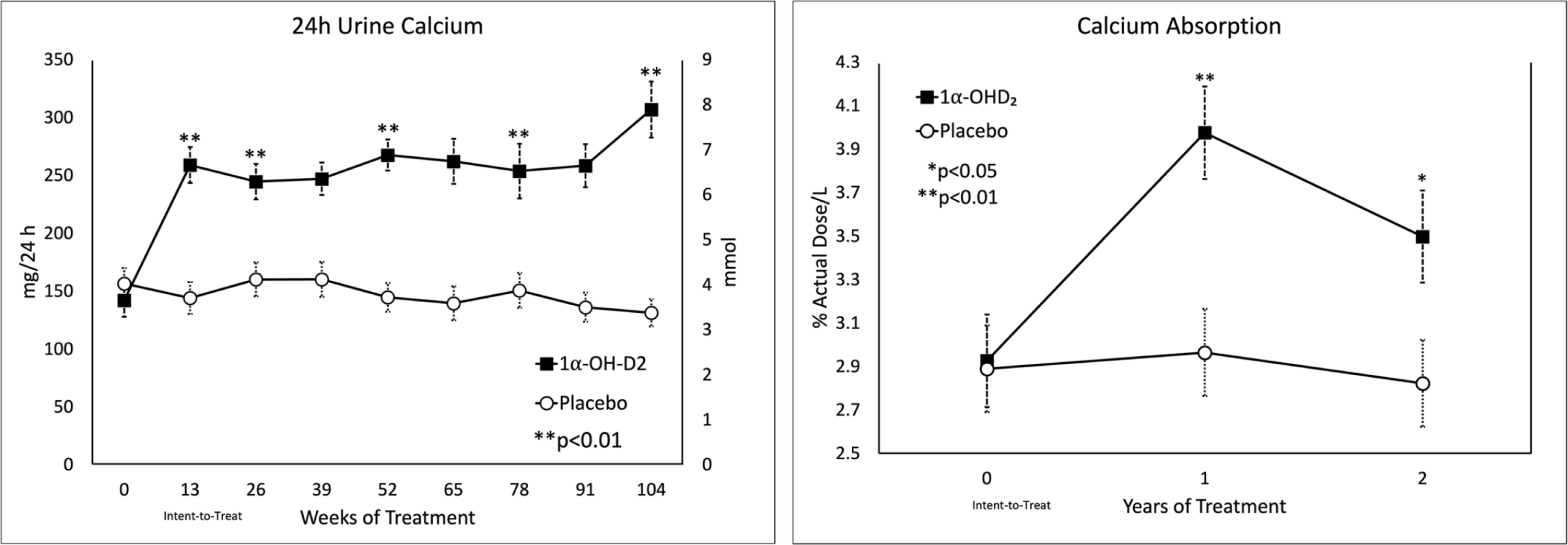
The 24-h urine calcium in doxercalciferol or placebo. Calcium absorption pre- and post-doxercalciferol. **p<0.01.

The 24-h urine calcium (in millimoles) in the doxercalciferol group increased from a baseline mean of 3.67 to 4.8 at week 3, to 6.12 at week 5, and to 6.67 at week 52. In year 2, the mean urine calcium levels were similar up to week 92, but the urine calcium averaged 7.67 in week 104. The placebo group averaged 4.15 at baseline, 3.62 at week 52, and 3.275 at week 104 (**Figure 2**). In the doxercalciferol group, 5 out of 28 (18%) subjects had episodes of hypercalciuria (>10 mmol) in year 1 and 6 out of 20 subjects in year 2, with the majority being single episodes and the highest urine calcium value being 10.75 mmol. In the placebo group, 1 out of 27 subjects had hypercalciuria in year 1, but 0 out of 21 in year 2. In subjects that never increased urine calcium >7.5 mmol (300 mg), the average baseline urine calcium was 2.1 mmol (84 mg). In subjects that increased urine calcium >7.5 mmol (300 mg), the average baseline value was 4.25 mmol (170 mg). In those that exceeded 10 mmol (400 mg), the baseline averaged 4.62 mmol (185 mg). Of 360 urine tests over 2 years, only 15 tests or 4% exceeded 10 mmol (400 mg).

In the doxercalciferol group, the calcium absorption at baseline averaged 65% (2.92% actual dose per liter), which increased to 79% on an average dose of 4.15 μg at 12 months and 72% on an average dose of 3.61 μg at 24 months. The corresponding values for the placebo group were 64% at baseline, 65% at 12 months, and 63% at 24 months. Statistical differences between groups were *p* < 0.001 at 12 months and *p* < 0.023 at 24 months (**Figure 2**).

#### 3.4.3 Creatinine clearance

There were no significant changes in creatinine clearance (in milliliters per minute) in either group over 24 months. In the doxercalciferol group, the creatinine clearance averaged 85.02 at baseline, 84.73 at 12 months, and 85.31 at 24 months. The creatinine clearance in the placebo group averaged 92.56 at baseline, 84.63 at 12 months, and 78.44 at 24 months (**Table 2**).

#### 3.4.4 Serum osteocalcin and PTH

The mean osteocalcin in the doxercalciferol group increased from 3.91 ng/ml at baseline to 4.07 ng/ml at 12 months and 4.34 ng/ml at 24 months. The mean in the placebo group at baseline was 3.88, at 12 months was 3.99, and at 24 months was 4.40 ng/ml. Differences between groups were not significant at any time (*p* = 0.6) (**Table 2**). The serum PTH (in picograms per milliliter) in the doxercalciferol group decreased from a mean baseline of 138 pg/ml to 110 pg/ml at 12 months and 145 pg/ml at 24 months. In the placebo group mean baseline was 117 pg/ml, 128 pg/ml at 12 months, and 176 pg/ml at 24 months (**Table 2**).

#### 3.4.5 Vitamin D metabolites

**Figure 3:**
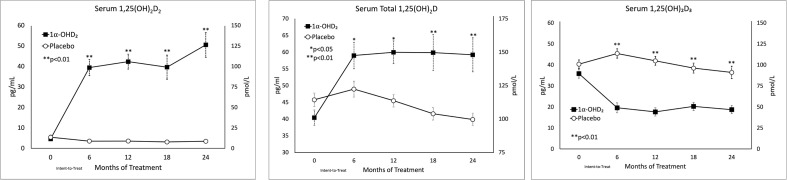
Changes in the serum vitamin D metabolites pre- and post-doxercalciferol. **p<0.01.

In the doxercalciferol group, the mean serum 25(OH)D at baseline was 75.13 nmol/L, but was 69.14 nmol/L at 12 months and 63.4 nmol/L at 24 months. In the placebo group, serum 25(OH)D averaged 73.63 at baseline, 73.88 at 12 months, and 67.14 at 24 months (**Table 2**). Serum 1,25(OH)_2_D (in picomoles per liter) (**Table 2**; **Figure 3**). The total serum 1,25(OH)_2_D in the doxercalciferol group increased from 100.79 to 148 at 12 months and 148.4 at 24 months compared with a decrease in the placebo group from 114 to 113.7 at 12 months and 99.4 at 24 months. The mean 1,25(OH)_2_D_2_ increased in the doxercalciferol group from the baseline of 11.4 to 105.6 at 12 months and 101.1 at 24 months (**Figure 2**). In contrast, the placebo group showed no increase, with 13.5 at baseline, 8.81 at 12 months, and 8.56 at 24 months (**Figure 3**). The mean serum 1,25(OH)_2_D_3_ decreased in the doxercalciferol group from 89.4 to 44 at 12 months and 46.4 at 24 months, while the values in the placebo group were relatively stable, with mean values of 100.7 at baseline, 104.6 at 12 months, and 90.8 at 24 months (**Figure 3**).

### 3.5 Bone mineral density results

**Figure 4:**
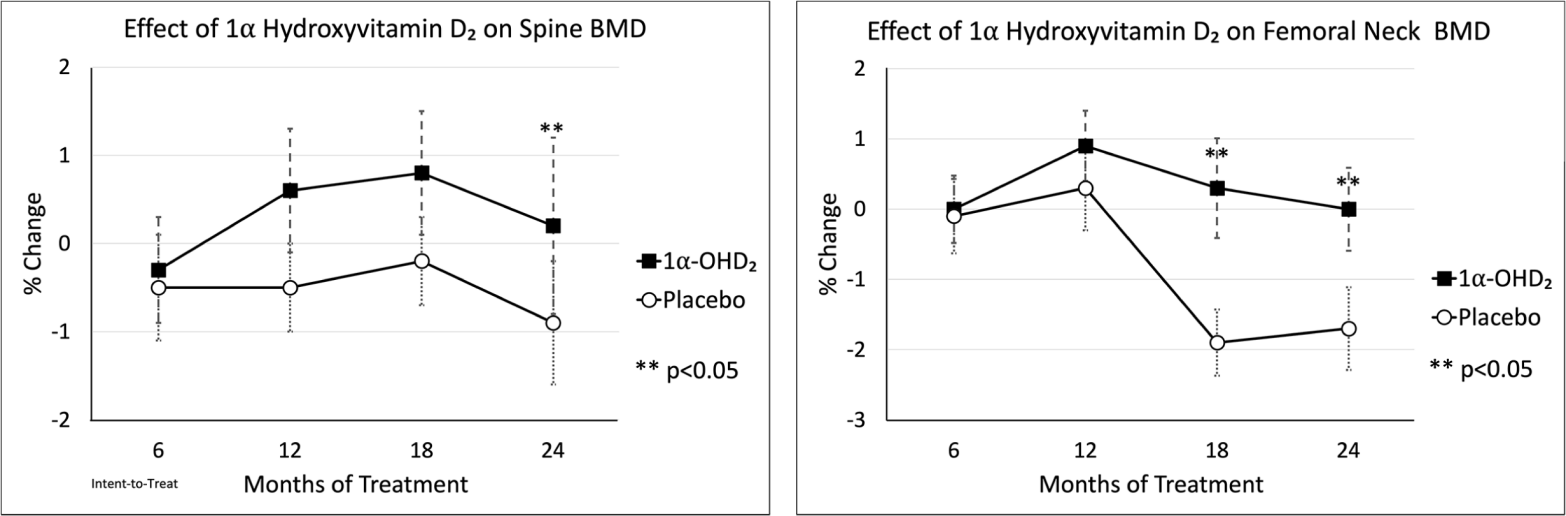
Changes in the spine and femoral neck bone mineral density (BMD) on treatment with doxercalciferol or placebo. **p<0.01.

All randomized patients were included in the ITT analysis. The results are expressed as percent change from baseline. In the PP analysis, three patients were omitted due to a high likelihood of primary hyperparathyroidism (two in doxercalciferol and one in placebo), and one patient was non-compliant on doxercalciferol as she had no measurable drug on multiple blood samples (**Figure 4**; **Table 2**).

In the ITT analysis of spine BMD, the doxercalciferol group increased 0.6% at 12 months and 0.2% at 24 months, while the placebo group decreased −0.5% at 12 months and −0.9% at 24 months, with a treatment/placebo difference of 1.1% (*p* = 0.36). Analysis of the doxercalciferol group showed that the spine BMD increased 1.1% at 12 months and 1.1% at 24 months, while that in the placebo group decreased −0.5% at 12 months and −1.3% at 24 months. The treatment/placebo difference was 1.6% at 12 months (*p* = 0.071) and 2.4% at 24 months (*p* < 0.041). In the ITT analysis of femoral neck BMD, the doxercalciferol group increased 0.9% at 12 months and 0.0% at 24 months; in the placebo group, the BMD increased 0.3% at 12 months and decreased −1.7% at 24 months (treatment/placebo difference = 1.7%, *p* < 0.047). In the PP analysis, the doxercalciferol group femoral neck BMD increased 0.7% at 12 months and 0.5% at 24 months, while in the placebo group the change was 0.2% at 12 months and −1.7% at 24 months. The treatment/placebo difference at 24 months was 2.2% (*p* < 0.017) (**Figure 4**).

### 3.6 Adverse events

There was a significant increase in urinary tract infections in the placebo group (*p* < 0.024). There were no reported clinical reports of renal stones during the study, and no calculi were seen in the urinary tract on radiographs at 12 or 24 months. Other medical adverse events were similar between the groups. As expected, there was a significant increase in the incidence of hypercalcemia (*p* < 0.020) and hypercalciuria (*p* < 0.001) on doxercalciferol.

### 3.7 General chemistry

There were no significant changes over 24 months in apparent safety in either of the two groups or in the between-group differences regarding the measurements of serum creatinine, serum phosphorus, serum glucose, BUN, the liver enzymes alanine aminotransferase (ALT; at 24 months) (**Table 3**) and aspartate aminotransferase (AST), bilirubin, alkaline phosphatase, serum albumin, cholesterol, triglycerides, hemoglobin, and the white blood cell differential counts. There were no significant changes in pulse rate and in the systolic and diastolic blood pressure in either group or between groups (**Table 2**).

**Table 3:** Baseline characteristics and changes at the end of year 1 and 2 on doxercalciferol /placebo mean T scores at baseline.

	Placebo BL	Placebo 1 year	Placebo 2 years	1α-OHD2 BL	1α-OHD2 1 year	1α-OHD2 2 years
	Mean (SD)	Mean (SD)	Mean (SD)	Mean (SD)	Mean (SD)	Mean (SD)
Serum glucose (mmol/L)	5.16 (0.44)	5.11 (0.5)	5.22 (0.62)	5.38 (0.61)	5.18 (0.52)	5.22 (0.62)
Serum blood urea nitrogen (mmol/L)	5.25 (1.29)	5.71 (1.25)	5.71 (1.35)	5.89 (1.86)	6.25 (1.79)	6.21 (1.36)
Serum alkaline phosphatase (μkat/L)	1.29 (0.28)	1.34 (0.41)	1.29 (0.36)	1.21 (0.23)	1.17 (0.24)	1.39 (0.3)
Serum ALT (μkat/L)	0.37 (0.25)	0.36 (0.17)	0.36 (0.14)	0.32 (0.13)	0.42 (0.27)	0.33 (0.11)
Serum AST (μkat/L)	0.41 (0.15)	0.4 (0.12)	0.38 (0.09)	0.35 (0.12)	0.40 (0.13)	0.37 (0.08)
Serum albumin (g/L)	44.5 (1.8)	44.6 (2.6)	45.7 (2.4)	45.1 (2.9)	45.3 (3.8)	45.4 (3.2)
Serum cholesterol total (mmol/L)	5.93 (1.01)	5.93 (0.98)	5.54 (0.92)	5.78 (0.89)	5.91(0.94)	5.65 (0.93)
Serum triglycerides (mmol/L)	1.46 (0.66)	1.48 (0.63)	1.46 (0.58)	1.51 (0.77)	1.59 (0.67)	1.53 (0.72)
Hemoglobin (g/dl)	141.4 (7.4)		141.9 (8.3)	137.3 (8.1)		138.1 (9.7)
White cell count (×10^9^)	58.1 (12.6)		57.7 (13.9)	58.6 (13.6)		55.3. (15)
Red blood cells (×10^3^)	4.523 (0.25)		4.67 (0.29)	4.423 (0.34)		4.609 (0.28)
Eosinophils (%)	2.4 (2.0)		3.3 (2.6)	2.9 (2.0)		2.6 (1.4)
Lymphocytes (%)	34.3 (8.6)		30.9 (7.9)	34.1 (9.1)		32.9 (9.3)
Monocytes (%)	4.8 (3.2)		3.7 (2.5)	6.0 (2.8)		4.5 (2.7)
Blood pressure systolic (mmHg)	123.2 (17.6)	124.7 (13.1)	122.7 (13.4)	126.6 (15.6)	127.4 (22.8)	124.8 (12.7)
Blood pressure diastolic (mmHg)	75.1 (8.9)	73.6 (6.3)	71.3 (7.4)	74.6 (9.0)	74 (6.9)	72.3 (5.6)
Pulse (per minute)	71.8 (6.3)	71.6 (8.1)	71.2 (7.3)	75.1 (9.7)	73.3 (6.3)	68.6 (7.3)
Intention to treat, ITT (*n*)	27	27	21	28	28	20
Per protocol, PP (*n*)	26	26	20	25	25	17

*BL*, baseline; *ALT*, alanine aminotransferase; *AST*, aspartate aminotransferase.

## 4 Discussion

Osteoporosis in women develops in two distinct phases: rapid loss due to decreased estrogen levels at the time of the menopause (44) and age-related bone loss that occurs at a slower rate for the remaining years after the menopause (45). A major contributing factor to age-related bone loss can be attributed to the development of intestinal malabsorption of calcium starting around age 65–70 years (4). Although the cause is not clear, there is certainly a decrease in the active transport of calcium in the duodenum and the upper ileum (46). Malabsorption of calcium is not due to a decrease in the level of the VDR measured on duodenal biopsies (47). However, a smaller study showed some lower values of VDR in a few of the older subjects (48). A later development after age 75–80 years, there is a 50% decline in serum 1,25(OH)_2_D due to declining renal function (5, 10).

Malabsorption of calcium in elderly subjects with osteoporosis is not normalized by treatment with simple vitamin D_2_ even in daily doses up to10,000 IU (9, 16), but is easily corrected by treatment with calcitriol 0.25 µg twice daily (11, 12). These results led to an interest in the effects of vitamin D on calcium metabolism and bone in patients with osteoporosis. Metabolic studies of calcium balance show that calcitriol 0.25 µg twice daily compared to placebo increased calcium absorption, reduced the rate of bone resorption measured on iliac crest biopsies and reversed negative calcium balance from -55mg/d to - 5mg/d after 6 months and -25 mg/d after one year (49). The use of vitamin D analogs, such as alfacalcidol, also increased the calcium absorption at a dose of 0.5 µg/day, whereas vitamin D_2_ at 1,000 IU/day had no effect (16).

In three placebo-controlled studies, the effect of calcitriol on BMD in osteoporosis was studied for 2 years using a dose escalation design. All subjects maintained calcium intakes of l,000 mg/day, while the calcitriol doses were increased up to 2 or to the point of toxicity, as indicated by hypercalcemia, and then reduced to the next lower dose until the serum calcium became normal. If hypercalciuria or hypercalcemia reoccurred, then the calcium intake was reduced to 600 mg/day. Two studies reported that calcitriol produced a significant positive effect on the total body calcium and spine BMD at long-term average dose of 0.7 µg (0.62 and 0.8 µg) (18, 20). The third study reported no difference between calcitriol and placebo therapy on spine BMD, but the average dose was only 0.4 µg, which is probably sub-physiologic. Reanalysis showed that the spine BMD increased in those who received doses above 0.5 µg (19). Of the subjects, 92% experienced hypercalcemia if the daily dose exceeded 1.0–2.0 µg, and all experienced hypercalciuria on a calcium intake of 1,000 mg. Between 0.5 and 1.0 µg, there was an increase in the incidence of hypercalcemia and hypercalciuria on a calcium intake of 1,000 mg, while a dose of 0.5 µg did not cause hypercalcemia or hypercalciuria if the calcium intake was restricted to 600 mg, thereby avoiding hyperabsorption at higher calcium intakes (18, 20).

Another vitamin D analog, eldecalcitol, showed a similar steep dose–response curve. On daily doses of 0.5, 0.75, and 1.0 µg, hypercalcemia occurred in 7%, 5%, and 23%, respectively (25). In an active comparator study with another vitamin D derivative, alfacalcidol (1α-hydroxyvitamin D_3_), the incidence of hypercalcemia was 21% on 0.75 µg eldecalcitol and 13.3% on 1 µg alfacalcidol. In these studies, the calcium intake was 700–840 mg/day (26). A comparison between calcitriol and alfacalcidol for calcium absorption and urine calcium showed a response on 0.14 µg calcitriol, but no effect of 0.65 µg alfacalcidol (50).

The potency of calcitriol and eldecalcitol on calcium absorption can cause hypercalciuria and hypercalcemia when the dietary calcium is high (800–1,000 mg) as the first-pass effect on intestinal absorption causes hyperabsorption of dietary calcium (11, 18–20, 50). Alfacalcidol does not have a first-pass effect on calcium absorption as it is not converted to 1,25(OH)_2_D until it is hydroxylated in the liver at the 25-position. This led us to study another 1α-hydroxylated derivative, i.e., 1α-OHD2 or doxercalciferol.

In an earlier dose-ranging study of doxercalciferol (Hectorol) lasting 6 weeks, doxercalciferol could be increased up to 5 µg daily with minimal effects on serum and urine calcium on a calcium intake of 600–800 mg (34). In the present long-term study, 5% of the subjects had an episode of very mild hypercalcemia (<10.7 mg/dl) and 20% had an episode of hypercalciuria; thus, doxercalciferol was less potent in causing hypercalcemia and hypercalciuria than calcitriol or eldecalcitol. In the present study, the average doses of doxercalciferol were 4.16 µg at 12 months and 3.61 µg at 24 months on an average calcium intake between 700 and 800 mg; thus, a similar effect on the bone can be obtained with a dose approximately seven times higher than calcitriol, three times higher than alfacalcidol, and six times higher than eldecalcitol, with fewer events of hypercalcemia and hypercalciuria. The advantages of doxercalciferol include its once daily dosing, flexibility in doses between 3 and 5 µg, and less hypercalcemia and hypercalciuria than calcitriol.

There are species differences in animal models, with vitamin D_2_ being less toxic than D_3_, and the median lethal dose for D_2_ is approximately 18 times higher than that for vitamin D_3_ (32). Vitamins D_2_ and D_3_ showed equal activity in the bones of rachitic rats and similar increases in calcium absorption and bone mobilization (51). In birds, there are differences in D_2_ *versus* D_3_ metabolism, and the apparent activity of 1,25(OH)_2_D_2_ is approximately 5–10 times lower than that of 1,25(OH)_2_D_3_, which has been attributed to the reduced binding of D_2_ to the transport protein that does not bind vitamin D compounds (52). In New World primates, vitamin D_2_ is less effective than vitamin D_3_ in the prevention of rickets (53), and there may be greater resistance to vitamin D_2_ than to calcitriol. On the other hand, in Old World primates, vitamins D_2_ and D_3_ have approximately equal biopotencies; however, very large doses of D_3_ cause fatal toxic effects, whereas equivalent doses of D_2_ are tolerated (54). In mice treated with equivalent doses of D_3_ or D_2_, bone mass increased significantly higher on D_2_ than on D_3_ probably because vitamin D_2_ binds with lower affinity than D_3_ to the vitamin D binding protein(DBP) resulting in higher free 25(OH)D_2_ levels that were twice as high as those of free 25(OH)D_3_ (55).

Surprisingly, the 1-alpha hydroxylated forms of D_2_ and D_3_ produced in the kidney appear to differ in their effects on bone and calcium. For example, 1α-OHD2 reduced the spine trabecular bone by 84% compared with 64% for 1α-OHD3 in ovariectomized rats; however, 1α-OHD3 increased the urinary calcium five times compared with two times for 1α-OHD2 (56). In a study of ovariectomized mice, doxercalciferol prevented bone loss, increased the markers of the bone formation markers Runx2, osterix, and Col1a1, and prevented cartilage damage (57). Patients with epilepsy on anticonvulsants, mainly phenobarbital and phenytoin, had decreased production of 1,25(OH)_2_D and more bone loss. Treatment with vitamin D_2_ or D_3_ (4,000 IU/day plus calcium 500 mg/day) for 6 months showed significant differences, with an increase in bone mass on D_2_ and no change on D_3_. Moreover, the urine hydroxyproline was reduced by 58% on D_2_ compared with 22% on D_3_, and the 24-h urine calcium/creatinine increased 90% on D_2_ and 142% on D_3_ (58). These active hydroxylated forms appear to differ in both effectiveness and safety. The positive skeletal effects of synthetic D_2_ analogs, along with their lower toxicity, have made both the prodrug doxercalciferol and the synthetic D_2_ analog paricalcitol beneficial in the treatment of patients with chronic renal disease, and both reduce secondary hyperparathyroidism due to calcitriol deficiency (59). In Cyp27B1 knockout (KO) mouse, doxercalciferol corrected both the elevated PTH and osteomalacia at 3–10 times lower doses than the paricalcitol analog (60).

It is not clear why it takes a long time for vitamin D analogs to increase BMD. This is not unique to this group of compounds. A bone remodeling cycle is somewhere between 6 and 12 months so that, in a 2-year study, there is time for only two to three cycles. In a study of high-dose calcitriol (2 µg daily for 6 months) in patients maintained on a low calcium intake of 500 mg daily, there was a remarkable anabolic response within 6 weeks on bone biopsies not seen on a dose of 0.5 µg daily (61). We believe that, in the first year, the early response is due to an anti-resorptive effect from increased calcium absorption, whereas by years 2–3 the long-term effect is due to an anabolic effect on osteoblasts, which occurs slowly because of the long remodeling sequence in bone cells. In a long-term study of eldecalcitol on bone, increases in serum osteocalcin and BMD occurred during year 3 and not in year 1 (25, 62).

Because a previous study of high-dose calcitriol of 2 µg daily with a low calcium intake of 500 mg showed a remarkable anabolic effect on the bone, it is possible that a much higher dose of doxercalciferol on a low calcium intake of 500 mg could show a larger anabolic effect on the bone. An active comparator trial of doxercalciferol *versus* alfacalcidol in the prevention of osteopenia and osteoporosis could show the relative risk benefit of these vitamin D compounds, both of which are in use today.

Although there are no long-term reports of renal impairment on calcitriol 0.5 µg daily, there have been reports of hypercalcemia and renal damage in the long-term follow-up of patients on the eldecalcitol 0.75-µg dose, particularly in patients older than 80 years (27). In an analysis of a medical database of 12,671 patients, more than half of the patients never had monitoring of serum calcium (28). The most likely cause of hypercalcemia is high calcium intake, and we recommend limiting the daily calcium intake to below 600 mg (11) and monitoring the serum calcium in patients exposed to other contributing factors with renal side effects, such as thiazides and non-steroidal anti-inflammatory drugs.

Doxercalciferol is already approved worldwide for the treatment of renal osteodystrophy and secondary hyperparathyroidism and is used in approximately half of all chronic kidney disease (CKD) patients. It is widely accepted as a useful agent with less toxicity than other analogs or metabolites (63). Long-term safety data, as demonstrated in the present study, further support its potential applicability in the management of additional disease states beyond mineral and bone disorders associated with CKD. Pediatric dialysis patients aged 15 years were treated with doxercalciferol, an average of 19 µg weekly for 8 months, and underwent iliac crest biopsies with *in vivo* histomorphometry and *in vitro* cell studies. Doxercalciferol improved mineralization and decreased osteoid tissue, and *in vitro* cellular studies suggest improved regulation of osteoclast–osteoblast coupling in CKD bone (64). In other studies, doxercalciferol was identified by molecular docking analysis to have the potential to reduce cartilage injury by decreasing the pro-inflammatory secretory phenotype in human chondrocytes. Doxercalciferol may suppress inflammatory mediators, including the Wnt-associated components (WACs), and potentially prevent the degradation of articular cartilage (65). There could be other potential uses for doxercalciferol, such as in the prevention of type 2 diabetes and reduction of the incidence of autoimmune diseases, respiratory infections, tuberculosis, coronavirus disease (COVID), and cancer.

## 5 Conclusions

The results of this pilot study showed that doxercalciferol prevents age-related bone loss at dose levels approximately seven times higher than that of calcitriol, four times higher than that of alfacalcidol, and five times higher than that of eldecalcitol, but with a lower incidence of hypercalcemia and hypercalciuria. Alfacalcidol 1 µg daily has been approved for the treatment of osteoporosis in Europe and Asia (22). However, there is a need for an active comparator trial of doxercalciferol *versus* alfacalcidol to assess whether doxercalciferol has any clinical advantage in the prevention and treatment of osteoporosis.

## Data Availability

The raw data supporting the conclusions of this article will be made available by the authors, without undue reservation.
